# Electrically tunable metasurface perfect absorber for infrared frequencies

**DOI:** 10.1186/s40580-017-0131-0

**Published:** 2017-12-21

**Authors:** Gwanho Yoon, Sunae So, Minkyung Kim, Jungho Mun, Renmin Ma, Junsuk Rho

**Affiliations:** 1 Department of Mechanical Engineering, Pohang University of Science and Technology (POSTECH), Pohang, 37673 Republic of Korea; 2 Department of Chemical Engineering, Pohang University of Science and Technology (POSTECH), Pohang, 37673 Republic of Korea; 3 School of Physics, Peking University, Beijing, 100080 China

**Keywords:** Electrically tunable metasurface, Perfect absorber, Indium-tin-oxide, Infrared, Drude model, Metal–oxide–semiconductor model

## Abstract

We theoretically investigate a metasurface perfect absorber based on indium-tin-oxide as active material. Our design scheme relies on conventional metal–oxide–semiconductor model and the Drude model. Inducing a voltage into the device causes a blue-shift of 50 nm in the reflectance spectrum in the infrared region. The total thickness of the device is only 3.5% of the working wavelength *λ* = 2.56 μm, and the rate of reflectance change reaches 5.16 at *λ* = 2.56 μm. Because the material that we use has advantages of easy fabrication and fast response, our design approach can be used for numerous applications on active plasmonic sensors and filters.

Metamaterials composed of subwavelength-scale artificial meta-atoms can have extraordinary optical properties that originate from the geometrical configuration of the meta-atoms, rather than from their chemical composition. Many exotic phenomena such as negative refraction, [1–3] super-resolution imaging, [4–6] perfect absorption [7] and optical cloaking [8, 9] have been demonstrated by controlling the shape of the meta-atoms.

Metasurface perfect absorbers (MPAs) are metamaterial-based devices that can perfectly absorb electromagnetic waves of certain wavelengths. Compared to conventional absorption devices based on multi-layered thin films, the MPA is very thin, usually less than one wavelength. The absorption spectrum of the MPA can be easily tuned by controlling the design of the meta-atoms, so the MPA has many possible practical applications such as plasmonic sensors and filters.

Tunability is an important goal of metasurface research, especially in MPAs. Research on tunable metasurfaces consider electrical, [10–18] thermal, [19] optical [20, 21] and mechanical [22–25] tuning methods. The electrical method exploits the effect of carrier concentration on the optical properties of materials; this approach controls the optical functionality of the device by applying a voltage. The thermal method exploits the effect of temperature on the phase of special materials such as VO_2_ and GeSbTe; the phase change induces change in the optical properties. The optical method changes the optical response of the materials by using photon energy to excite electrons from a valence band into a conduction band; this process is fast, but independent and simultaneous control of each meta-atom is a difficult task. The mechanical methods are based on direct transformation of the physical profile of the meta-atoms; these methods include microelectromechanical systems (MEMS). Changing the shape of the meta-atom can generate a huge variation of resonance characteristics, but its working frequency is relatively low, because MEMS technology works on a scale of micrometers.

Electrically tunable metasurfaces (ETMs) are the most promising for practical application, because they are compatible with complementary metal-oxide semiconductors (CMOSs). ETMs use voltage as a power source, so they can be integrated into conventional semiconductor devices. A typical ETM is based on active materials such as indium-tin-oxide (ITO), GaAs or graphene. Of these materials, ITO is the cheapest; it is also easy to deposit on any kind of substrates, by either conventional electron beam evaporation or sputtering. Furthermore, the charge-carrier concentration ρ of ITO can be controlled from 10^19^ to 10^21^ cm^−3^ by annealing it under controlled conditions.

In this work, we propose an ITO-based electrically-tunable metasurface perfect absorber (ETMPA) that works near wavelength *λ* = 2.56 μm. Our device has a metal–insulator–metal (MIM) structure with square patch antennas to maximize the absorbance. Complex permittivity of ITO is derived using the Drude model, and the effect of external voltage on carrier concentration according is calculated using the conventional metal–oxide–semiconductor (MOS) model. The maximum absorbance reached 98% near *λ* = 2.56 μm, and the absorption spectrum blue-shifted by 50 nm as the external voltage was increased from 0 to 9 V. The rate of reflectance change reached 5.16 at *λ* = 2.56 μm. Our device and design approach have possible applications in plasmonic sensors and in active devices based on ETMPA.

Our device design has MIM structure that consists of a bottom aluminum (Al) mirror, an alumina (Al_2_O_3_) spacer and an Al metasurface on a glass substrate (Fig. 1). Al is highly reflective in the IR and is therefore an appropriate structural material. ITO thin film as the active layer is inserted between the metasurface and the spacer. When a voltage is applied between ITO and bottom Al, free electrons in the ITO layer accumulate near the interface of ITO and Al_2_O_3_. The device is very thin, i.e., ~ *λ*/28. The insulator has an important influence on the strength of electric field induced by the voltage bias. Al_2_O_3_ has high relative permittivity (9.3 ≤ *κ* ≤ 11.5) so a strong electrical field can be generated inside the insulator; hence, the carriers can accumulate strongly.

**Fig. 1:**
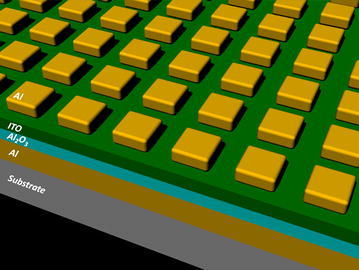
Schematic of the proposed device. Thickness are: bottom Al mirror, 40 nm; Al_2_O_3_, 15 nm, ITO layer, 15 nm. Top metasurface is composed of square patch nanoantennas that are 20 nm thick with side length of 450 nm, and are set at a pitch of 600 nm. Electrodes are connected between bottom Al and ITO layer to generate an electric field inside the insulator. The incident direction of the light is perpendicular to the surface

The carrier accumulation process can be described by the MOS structure, which is a basic model of the MOS field-effect transistor. In this work, ITO is regarded as a semiconductor because its intrinsic carrier density is similar to that of semiconductors. When the electric field is generated between ITO and the bottom Al mirror, free electrons in the ITO layer move toward the interface between ITO and Al_2_O_3_ (Fig. 2), whereas free electrons in bottom Al move away from interface the between insulator and bottom Al; the combination of these processes is called depletion. The number of free electrons is much larger in metal than in ITO, so carrier depletion in the bottom Al is negligible.

**Fig. 2:**
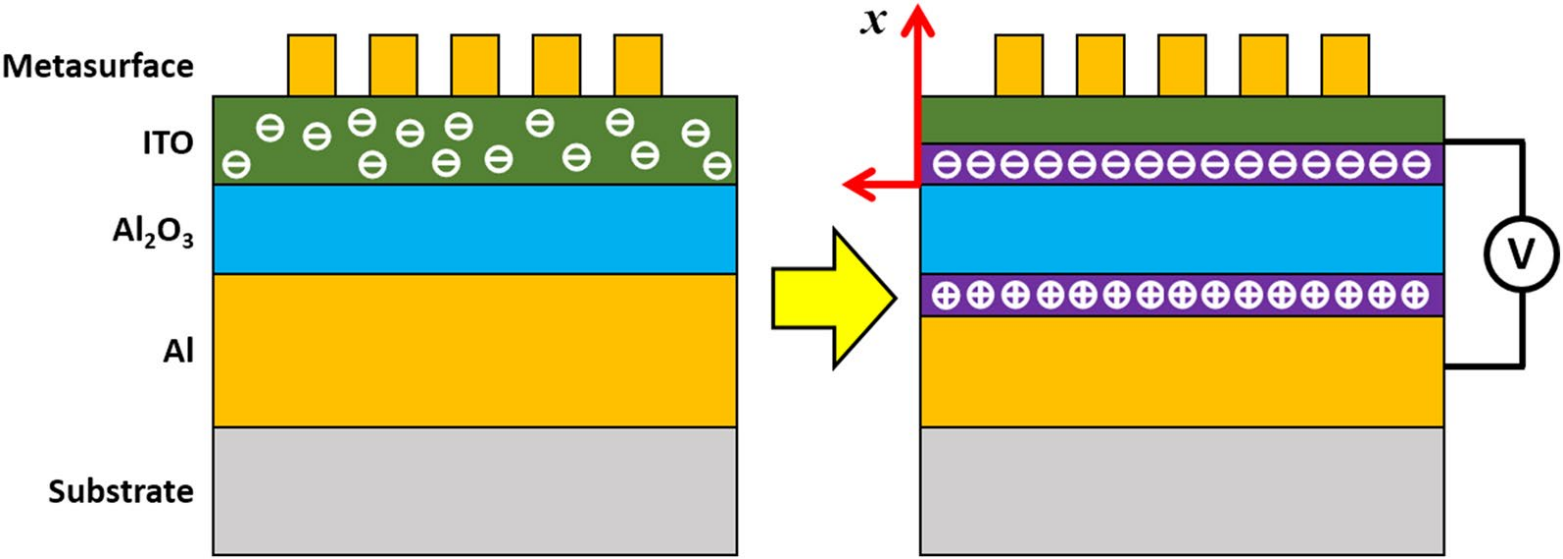
Illustration of carrier movement inside ITO under voltage bias. Free electrons in the ITO layer accumulate near the interface of ITO and Al_2_O_3_. Accumulated carrier concentration decreases exponentially as distance from the interface increases. Direction x represents a reference coordinate to describe carrier density along to the depth of the ITO layer in Fig. 3

Poisson’s equation with some appropriate assumptions gives the potential in the ITO as [26]

1 $$\varPhi \left( x \right) = - \frac{kT}{q}ln\left\{ {\sec^{2} \left[ {C - \frac{x}{{\sqrt 2 L_{D} }}} \right]} \right\},$$

where *x* [m] is the distance from the interface (Fig. 2), *k* = 8.617 × 10^−5^ eV/K is the Boltzmann constant, *T* [K] is temperature and *q* = 1.609 × 10^−19^ C is the electron charge, and

2 $$L_{D} = \sqrt {\frac{{\varepsilon_{ITO,\infty } \varepsilon_{0} kT}}{{q^{2} N_{d} }}} ,$$

[m] is the Debye length, where *ε*_*ITO,∞*_ = 3.74 [12] is infinite frequency relative permittivity of ITO, *ε*_*0*_ = 8.854 × 10^−12^ F/m is vacuum permittivity, and *N*_*d*_ [m^−3^] is the intrinsic carrier concentration of ITO. The constant *C* is related to surface potential *Φ*_*S*_ [V] as

3 $$C = \cos^{ - 1} \left( {exp\left[ {\frac{{q\varPhi_{S} }}{2kT}} \right]} \right).$$

*Φ*_*S*_ can be obtained from

4 $$V = \varPhi_{S} - \frac{{\varepsilon_{ITO,\infty } }}{{\kappa_{OX,\infty } }}t_{OX} \frac{\sqrt 2 }{{L_{D} }}\frac{kT}{q}\sqrt {exp\left( { - \frac{{q\varPhi_{S} }}{kT}} \right) - 1}$$

where *κ*_*OX,∞*_ = 11.1 is infinite frequency relative permittivity, and *t*_*OX*_ = 15 nm is the thickness of the insulator.

Finally, the carrier concentration *N*(*x*) [m^−3^] at distance *x* [m] from the interface according to the potential inside ITO can be calculated as

5 $$N\left( x \right) = N_{d} exp\left[ {\frac{q\varPhi (x)}{kT}} \right].$$

If no external voltage is applied, *N*(*x*) = *N*_*d*_. When voltage is applied, *N*(*x*) is maximized at the interface and decreases exponentially as *x* increases (Fig. 3). The ITO layer in our device has *N*_*d*_ = 10^19^ cm^−3^, and the maximum value of *N*(*x*) is increased to 1.2 × 10^22^ cm^−3^ by inducing a voltage of 9 V.

**Fig. 3:**
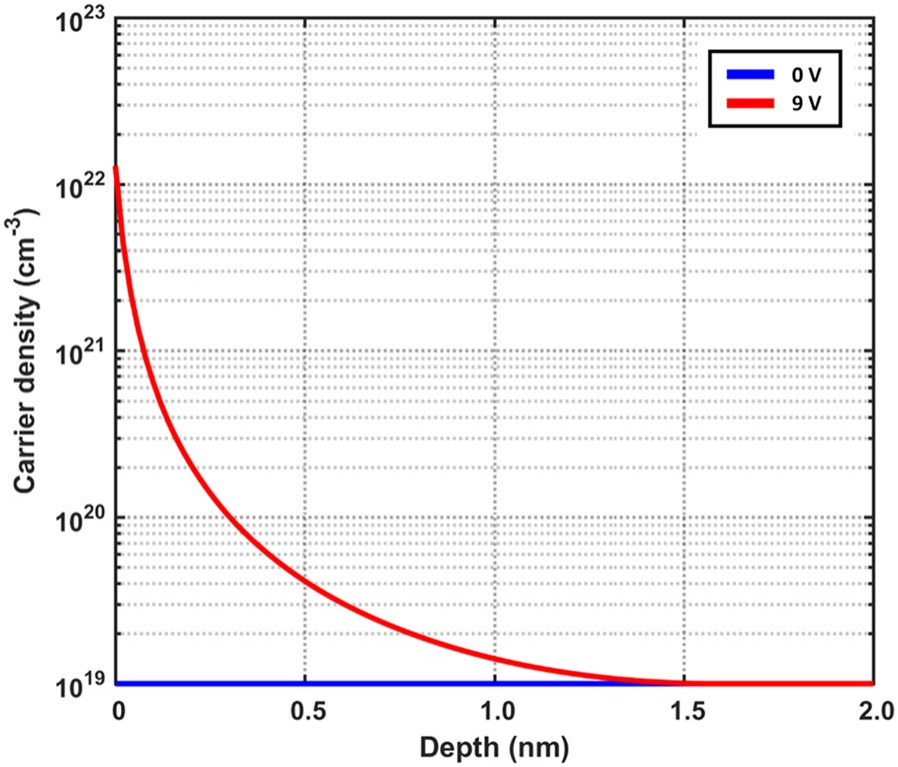
Carrier density distribution inside the ITO layer based on the MOS model. In calculation, the intrinsic carrier concentration of ITO is assumed 10^19^ cm^−3^ (blue line) while 9 V of voltage bias causes the concentration to increase to 1.2 × 10^22^ cm^−3^ near the interface (red line)

*N*_*d*_ is an important factor that determines the maximum *N*(*x*) as well as the accumulation thickness. As *N*_*d*_ increases, the maximum *N*(*x*) increases, but the accumulation thickness decreases which explains why the accumulation effect in metal can be ignored. Therefore, *N*_*d*_ of the ITO layer should be optimized to maximize the functionality of devices that use it.

The complex permittivity *ε* of ITO is strongly related to *N*_*d*_, so we can derive *n* by using the conventional Drude model. Although the Drude model is usually used to describe metals, it is applicable to ITO, because it has numerous free electrons. The dispersive permittivity of ITO is expressed as

6 $$\varepsilon (\omega ) = \varepsilon_{ITO,\infty } - \frac{{q^{2} N}}{{\varepsilon_{0} m_{e}^{*} }}\left( {\frac{1}{{\omega^{2} + i\gamma \omega }}} \right),$$

where and *ω* [rad/s] is the angular frequency of the incident wave. $m_{e}^{*}\,=\,0.45\,m_{e}$  is the effective electron mass in ITO (where *m*_*e*_ [kg] is electron mass in free space), and *γ* = 10^14^ rad/s is the collision frequency of ITO [27].

Based on the Eq. (6), complex permittivity of ITO is drawn according to the various carrier concentrations (Fig. 4). The real part of the permittivity decreases to negative values as the carrier concentration increases, and this can be understood as ITO becomes metallic. The imaginary part increases as the carrier concentration increases. In the target wavelength region from 2 to 3 μm, permittivity variation becomes larger as the wavelength increases.

**Fig. 4:**
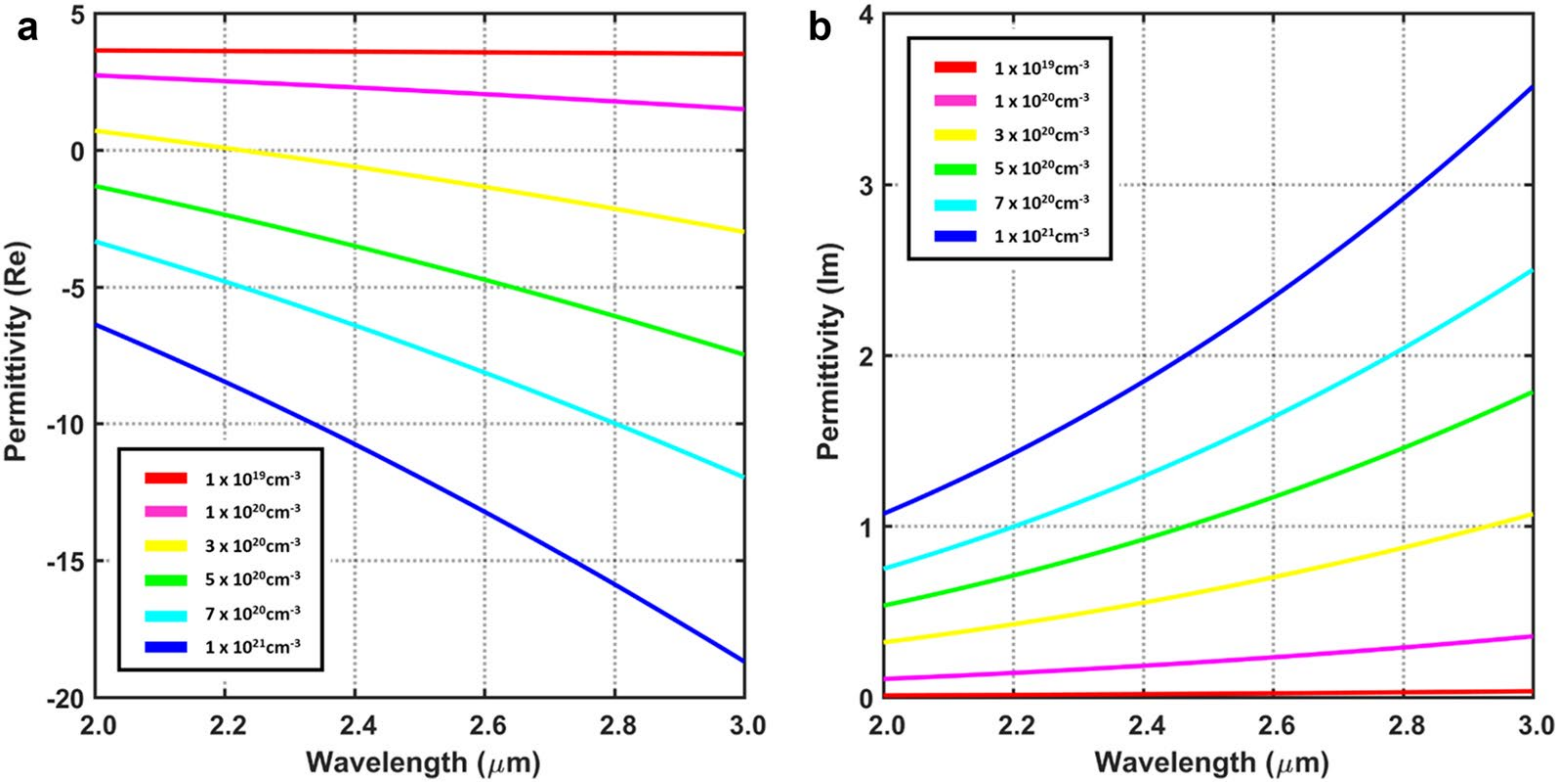
Calculated permittivity of ITO according to the different carrier concentration from 10^19^ to 10^21^ cm^−3^, by Drude modelling of ITO. When voltage was applied to the device, **a** the real and **b** imaginary parts of the complex permittivity vary concurrently and in opposite directions

By using COMSOL software, reflectance spectra depending on the different voltage bias are obtained based on the permittivity calculation data (Fig. 5). In order to reflect the exponentially decaying carrier density profile in the accumulation layer, mesh size is set to around 0.2 nm which is much less than accumulation thickness of ~ 1.5 nm. Without voltage bias, minimum reflectance is ~ 2% at *λ* = 2.56 μm, and it moves to *λ* = 2.51 μm according to the 9-V voltage bias having the reflectance of ~ 10%. We also calculate the rate of change which is defined as reflectance variation between 0 V and 9 V divided by unbiased reflectance (0 V). The maximum rate reaches up to 5.16 near *λ* = 2.56 μm. Negative rate means that the reflectance decreases as we induce the voltage bias. Our metasurface pattern is an array of square patch antennas, so the reflectance does not change regardless of incident polarization direction (Fig. 5d). Reflectance simulation on polarization angles is conducted with incident angles from 0° to 45°, but it is enough to cover from 0° to 360° because of the symmetry of our metasurface pattern design.

**Fig. 5:**
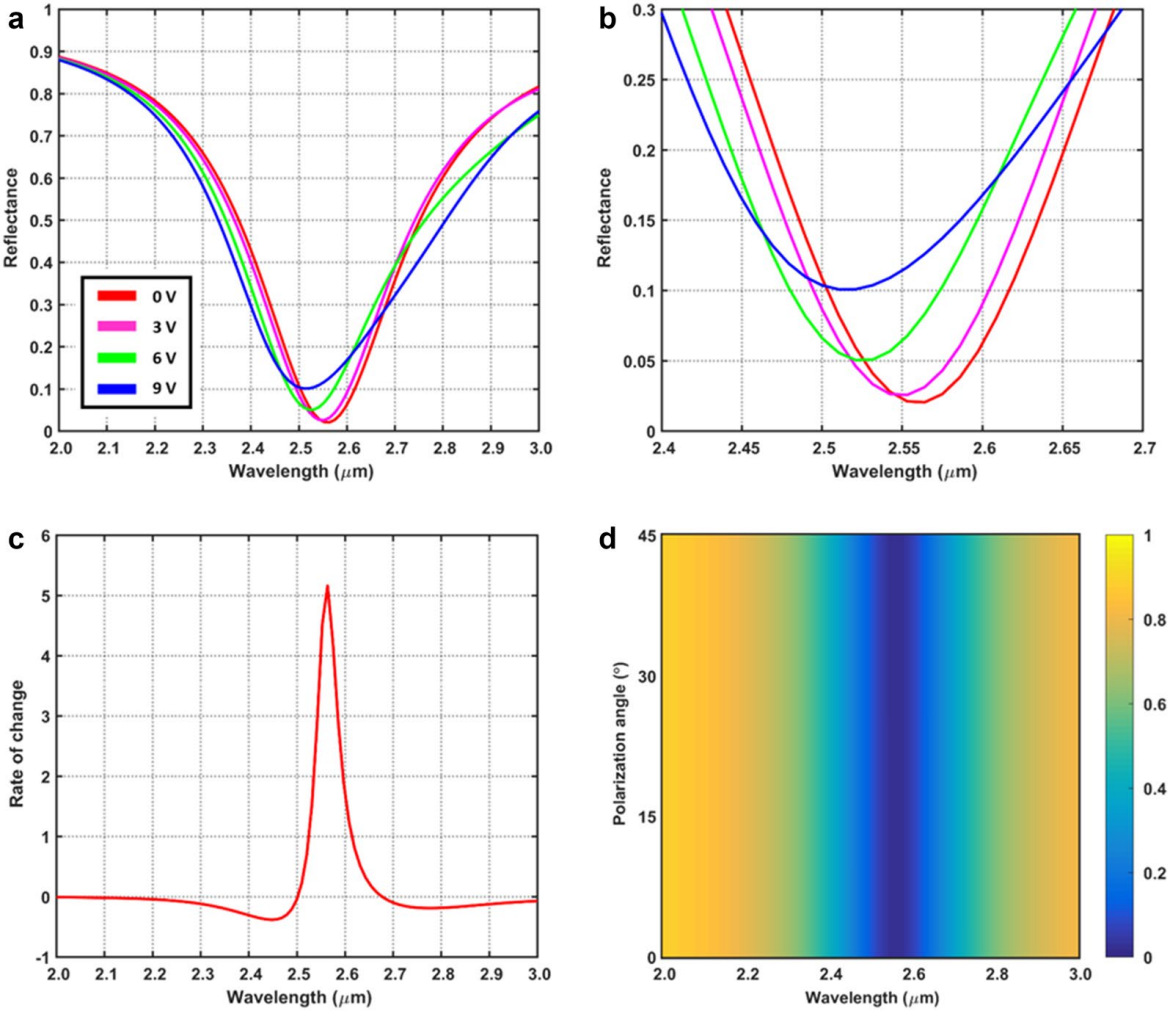
COMSOL simulation results of device functionality. **a** Reflection spectra of the device at voltage bias = 0, 3, 6 and 9 V. The minimum of reflection was near *λ* = 2.56 μm at 0 V, and moved 50 nm to the blue at 9 V. **b** Magnified plot near *λ* = 2.56 μm. **c** Calculated rate of reflectance change based on (**a**). The rate is defined by the reflectance variation divided by the unbiased reflection at each *λ*, which reaches up to 5.16 near *λ* = 2.56 μm. **d** Calculated unbiased reflectance result depending on the incident polarization direction from 0° to 45°. The reflectance seldom changes regardless of polarization direction because of the symmetry of our metasurface pattern design

In summary, an ETMPA that works in the IR region is presented. Carrier concentration inside the ITO layer is analyzed based on the conventional MOS model, and the corresponding complex permittivity of ITO is derived using Drude modelling. When bias is applied to the device, the minimum point of the reflectance spectrum blue-shifts by 50 nm, and the rate of reflectance change reaches 5.16 at *λ* = 2.56 μm. Overall thickness of the device is only *λ*/28. Our design scheme based on ITO can be used to convert passive metasurface devices to active devices. Compared to other tuning mechanisms, our method has advantages of CMOS compatibility and compact size. We believe that our approach provides an intuition to researchers who try to develop active devices based on metasurfaces and plasmonics, such as absorbers, reflectors, sensors and filters.
